# Differential temporal changes of primary and secondary bacterial symbionts and whitefly host fitness following antibiotic treatments

**DOI:** 10.1038/srep15898

**Published:** 2015-10-29

**Authors:** Chang-Rong Zhang, Hong-Wei Shan, Na Xiao, Fan-Di Zhang, Xiao-Wei Wang, Yin-Quan Liu, Shu-Sheng Liu

**Affiliations:** 1 Ministry of Agriculture Key Laboratory of Agricultural Entomology, Institute of Insect Sciences, Zhejiang University, Hangzhou 310058, China

## Abstract

Where multiple symbionts coexist in the same host, the selective elimination of a specific symbiont may enable the roles of a given symbiont to be investigated. We treated the Mediterranean species of the whitefly *Bemisia tabaci* complex by oral delivery of the antibiotic rifampicin, and then examined the temporal changes of its primary symbiont “*Candidatus* Portiera aleyrodidarum” and secondary symbiont *“Ca.* Hamiltonella defensa” as well as host fitness for three generations. In adults treated with rifampicin (F0), the secondary symbiont was rapidly reduced, approaching complete disappearance as adults aged. In contrast, the primary symbiont was little affected until later in the adult life. In the offspring of these adults (F1), both symbionts were significantly reduced and barely detectable when the hosts reached the adult stage. The F1 adults laid few eggs (F2), all of which failed to hatch. Mating experiments illustrated that the negative effects of rifampicin on host fitness were exerted via female hosts but not males. This study provides the first evidence of differential temporal reductions of primary and secondary symbionts in whiteflies following an antibiotic treatment. Studies that disrupt functions of bacterial symbionts must consider their temporal changes.

Associations with symbiotic microorganisms, especially bacteria, are pervasive in insects and other terrestrial arthropods^1^. Resident microorganisms in insects, often transmitted with high fidelity from mother to offspring, exert a range of substantial impact on the ecology and evolution of the hosts^2^,^3^,^4^,^5^,^6^,^7^. In aphids, all species of this group of plant phloem feeding insects examined to date harbour the primary, obligate symbiont, “*Candidatus* Buchnera aphidicola”, which plays a critical role in nutrient provision^8^,^9^,^10^. In addition, each species of aphids may have one or more secondary, facultative symbionts that modulate ecologically important traits of the hosts^11^. Experiments with the pea aphid, *Acyrthosiphon pisum*, have demonstrated that facultative symbionts may protect the host from attacks by natural enemies^12^,^13^,^14^,^15^, ameliorate harmful effects of heat^16^,^17^, alter host colour^18^, and modulate suitability of host plants^19^,^20^. Not surprisingly insect symbiosis has attracted more and more attention from scientists as well as pest management practitioners; see review by Douglas (2015)^10^.

The whitefly, *Bemisia tabaci* (Gennadius) (Hemiptera: Aleyrodidae), is a species complex including at least 34 morphologically indistinguishable species^21^,^22^,^23^. The Middle East-Asia Minor 1 (MEAM1) and Mediterranean (MED) species, formerly often referred to as the B and Q “biotypes”, of the whitefly complex are notorious invasive pests and have caused enormous losses to agriculture worldwide in the past 30 years^24^. Like other phloem-feeding insects, whiteflies need the primary symbiont “*Candidatus* Portiera aleyrodidarum” to provide essential amino acids and carotenoids that are limited or lacking in the phloem sap^25^,^26^,^27^,^28^. In addition, seven genera/species of secondary symbionts, namely “*Candidatus* Hamiltonella defensa”, “*Candidatus* Wolbachia spp.”, *Arsenophonus* spp., “*Candidatus* Cardinium hertigii”, “*Candidatus* Fritschea bemisiae”, *Rickettsia* sp., and “*Candidatus* Hemipteriphilus asiaticus” have been reported from this whitefly species complex^29^,^30^,^31^,^32^. Different species of the *B. tabaci* complex often harbour different secondary symbionts^32^,^33^,^34^,^35^,^36^,^37^. In the populations of various *B. tabaci* cryptic species maintained in our laboratory, the secondary symbionts varied in species and composition; the MED whitefly tested in this study harbours only *Hamiltonella*^36^.

In the last 10 years much research has focused on the functions of symbionts in the biology of the *B. tabaci* species complex. For example, *Rickettsia* spp. in MEAM1 have been reported to enhance host fitness and female sex ratio^38^, host thermal tolerance^39^, host resistance against parasitoids^40^ and entomopathogenic bacteria^41^, and host capacity to transmit viruses^42^ as well as host susceptibility to insecticides^43^. Like all the symbionts found in aphids, those detected in whiteflies cannot be cultivated. As in studies of aphid symbiosis, experimental examinations of the functions of symbionts in whiteflies have been mostly conducted by comparing the performance between whitefly lines of similar genetic background bearing or lacking a given symbiont^38^,^44^,^45^,^46^,^47^,^48^. Such experimental lines are often created by introgression^38^, antibiotic treatment of the hosts to selectively cure one or more facultative symbionts^44^,^45^,^46^,^47^,^48^, or microinjection to transfer a given symbiont between host populations^49^,^50^. These attempts to establish such experimental whitefly lines vary widely. In particular, attempts to use antibiotic treatments to cure certain symbionts range from no success^51^ (Dr. Murad Ghanim, Agricultural Research Organization, Israel, email: ghanim@volcani.agri.gov.il, personal communication; Dr. Einat Zchori-Fein, Agricultural Research Organization, Israel, email: einat@volcani.agri.gov.il, personal communication; Professor Martha S. Hunter, University of Arizona, USA, email: mhunter@ag.arizona.edu, personal communication), partial success^44^,^48^, to complete successes^45^,^46^,^47^.

Studies on the functions of uncultivable microbes, even with modern molecular and bioinformatics techniques, still face enormous challenges^52^. Establishing experimental insect lines lacking or bearing certain symbionts has proved to be a powerful tool in gaining an understanding of the biological roles of individual symbionts in complex systems where an insect harbours multiple endosymbiotic bacteria^11^,^53^,^54^,^55^. We too have attempted to establish lines of a given whitefly species bearing or lacking certain symbionts via antibiotic treatments in the past four years. Here we report the results of antibiotic treatment of the MED whitefly, which bears the primary symbiont *Portiera*, and only one secondary symbiont, *Hamiltonella*^36^. First, we treated whitefly adults with the antibiotic rifampicin at various concentrations to select an appropriate dose for symbiont curing. Next, we monitored whitefly performance and temporal dynamics of symbiont density through several generations following rifampicin treatment. Finally we conducted mating experiments between rifampicin-treated and untreated whiteflies to assess whether the effects of rifampicin treatment on fitness of the whitefly were exerted through both sexes or through only females as the symbionts are transmitted maternally^31^. Our study provides the first set of data to demonstrate the differential temporal reductions of primary and secondary symbionts in whiteflies following an antibiotic treatment, and illustrates that the antibiotic exerted its effects on host fitness via females and whether the males are treated has little contribution. Significantly, we show it is not feasible to cure the secondary symbiont by this antibiotic treatment without affecting the primary symbiont and establish MED whitefly lines lacking *Hamiltonella* for experimental studies.

## Results

We fed newly emerged whitefly adults in a glass feeding chamber for 48 h with a diet containing rifampicin to exercise the antibiotic treatments. These adults we designate as F0, and their progeny as F1and then the progeny of F1 as F2.

### Frequencies of curing symbionts of whiteflies

Symbiont curing by rifampicin in F0 adults was conducted at five concentrations from 1–100 μg/ml (Table 1). Immediately after feeding on the rifampicin-treated diet for 48 h, both the primary symbiont *Portiera* and the secondary symbiont *Hamiltonella* were undetected in a small proportion of individuals in each of the treatments, and the relative frequencies of curing appeared to vary among the five concentrations of rifampicin, but none differed significantly from the control (Table 1). Thirteen days after feeding on the rifampicin-treated diet, the frequencies of curing for the primary symbiont ranged from 3/22 to 8/22, and those for the secondary symbiont ranged from 18/22 to 20/22; the relative frequencies of curing for the secondary symbiont were markedly higher than those for the primary symbiont. Thirty days after the F0 adults fed on the rifampicin-treated diet, their progeny (F1) had developed to adults, and these adults were then tested for the presence of the symbionts. In four treatments both the primary and secondary symbionts were not detected (Table 1). In the 1 μg/ml rifampicin treatment, in two out of 22 adults we could still detect the presence of the secondary symbiont (Table 1).

### Whitefly survival and temporal dynamic of symbiont density after rifampicin treatment

**Figure 1:**
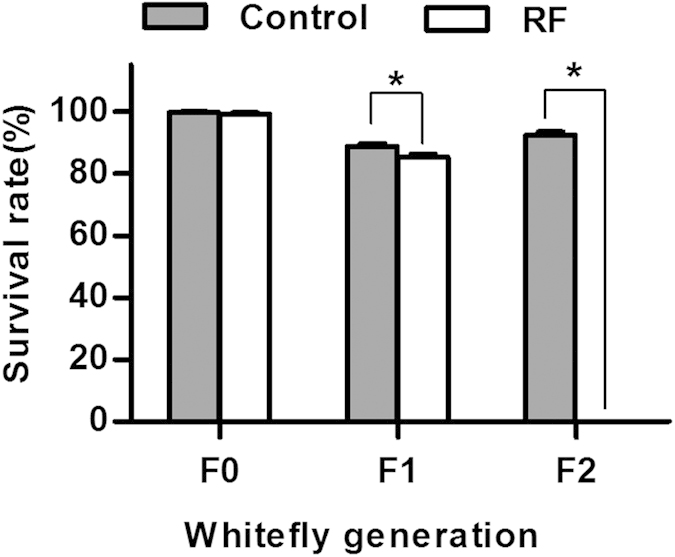
Effects of rifampicin treatment on survival of F0 adults after 48 h feeding on rifampicin-treated diet, survival of F1 whiteflies from egg to adult, and survival of F2 whiteflies from egg to adult. The asterisk indicates significant difference between rifampicin treatment (RF) and control within each of the generations based on Independent-Sample *t* test (*P* < 0.05).

After 48 h feeding on a diet with 30 μg/ml of rifampicin, the survival of the F0 whiteflies was little affected and did not differ from that of the control, and the survival of F1 from egg to adult was only marginally reduced compared to that of the control (Fig. 1). However, all the F2 eggs in the rifampicin treatment failed to hatch, while the F2 whiteflies in the control achieved a mean survival of 92% from egg to adult (Fig. 1).

**Figure 2:**
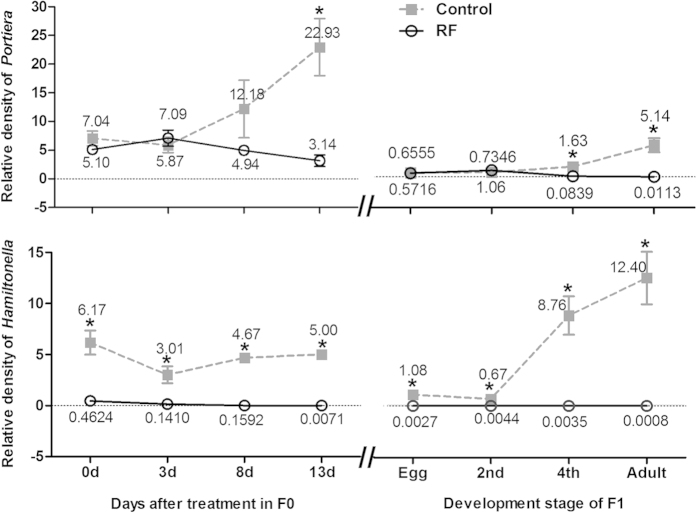
Temporal dynamics of *Portiera* (upper panel) and *Hamiltonella* (lower panel) in whiteflies after rifampicin treatment. The changes of symbiont density were detected at different time points of adult stage in F0 whiteflies and at different developmental stages in F1 whiteflies. *Portiera* and *Hamiltonella* were measured in terms of the number of 16S rRNA gene copies per *β-actin* gene copy, and mean values are shown. The asterisk indicates significant difference between rifampicin treatment (RF) and control at a given same time point or development stage based on Mann-Whitney test at α = 0.05.

Relative densities of the primary symbiont *Portiera* and secondary symbiont *Hamiltonella* showed different patterns of change after rifampicin treatment. In F0, the relative density of the primary symbiont of the whitefly adults did not differ significantly from that of the control until 13 days after treatment when a significant reduction was observed; in contrast, the relative density of the secondary symbiont was markedly reduced immediately after the 48 h feeding on the rifampicin-treated diet and was approaching zero by day 13 (Fig. 2, panels on the left). The relative density of the primary symbiont in F1 whiteflies in the rifampicin treatment did not differ from that of the control at egg and 2^nd^ instar, but was significantly reduced in comparison to that of the control at the 4^th^ instar and adult stage; in contrast, the secondary symbiont in the rifampicin treatment was barely detectable at each of the four host stages while all individuals in the control were found to harbour the secondary symbiont at each of the four stages examined (Fig. 2, panels on the right).

### Adverse effects of rifampicin treatment on whitefly performance in relation to host sex

**Figure 3:**
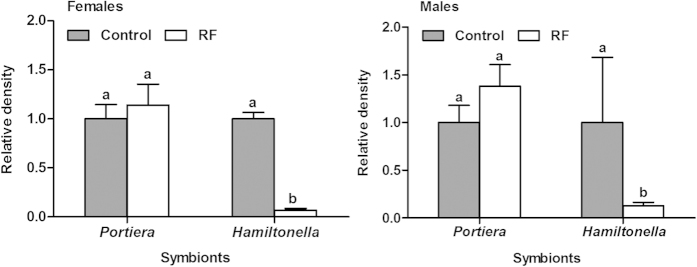
Relative densities of *Portiera* and *Hamiltonella* of female and male F0 whiteflies used in the mating experiment. The relative density of each of the two symbionts in the control was set to 1 (grey bar). Different letters above the bars of each symbiont indicate significant difference based on Mann-Whitney test at α = 0.05 between rifampicin treatment (RF) and control.

**Figure 4:**
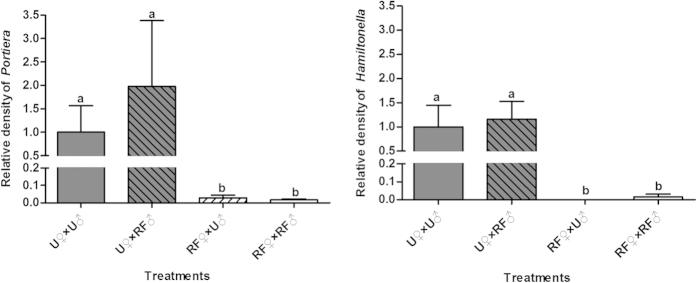
Relative densities of *Portiera* and *Hamiltonella* of female F1, i.e., offspring produced by F0 adults in the mating experiment. RF: rifampicin treated whiteflies; U: untreated whiteflies. Different letters above the four bars in each symbiont indicate significant difference between the four treatments based on Kruskal-Wallis test at α = 0.05 (*P* < 0.05).

Following rifampicin treatment, in both females and males of the F0 adults the primary symbiont was not significantly reduced whereas the secondary symbiont was reduced significantly compared to those of untreated, control adults (Fig. 3). Compared to the control treatment (U♀ × U♂), the number of eggs produced in 48 h after the antibiotic treatment was not significantly reduced in crosses that involved rifampicin-treated adults, either male (U♀ × RF♂) or female (RF♀ × U♂), or both sexes (RF♀ × RF♂). The performance of the progeny of treatment U♀ × RF♂, in which only males received rifampicin treatment, did not differ from that of the control U♀ × U♂ either (Table 2). However, compared to the control, the performance of F1 whiteflies in both treatments RF♀ × U♂ and RF♀ × RF♂ was significantly reduced in terms of eggs hatching and development time of subsequent immatures (Table 2). Similarly, the relative densities of both *Portiera* and *Hamiltonella* in F1 female adults of treatment U♀ × RF♂ did not differ from those of the control U♀ × U♂; whereas both symbionts in the F1 females of treatments RF♀ × U♂ and RF♀ × RF♂ were hardly detected (Fig. 4).

**Figure 5:**
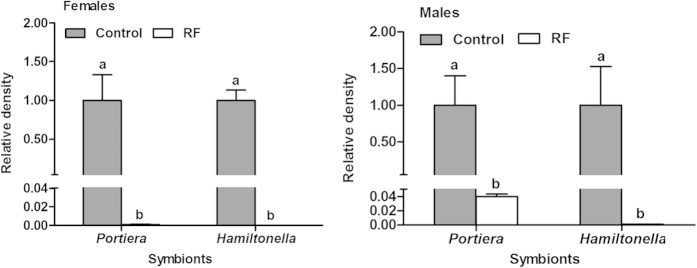
Relative densities of *Portiera* and *Hamiltonella* of female and male F1 whiteflies used in the mating experiment. The relative density of each symbiont in control was set to 1 (grey bar). Different letters above the bars of each symbiont indicate significant difference based on Mann-Whitney test at α = 0.05 between rifampicin treatment (RF) and control.

**Figure 6:**
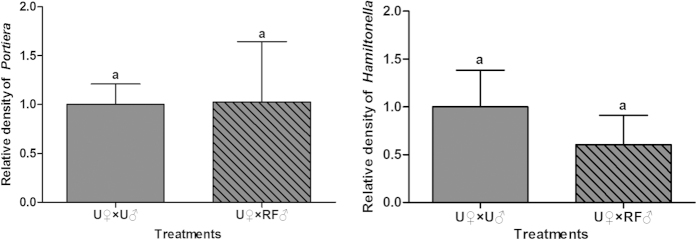
Relative densities of *Portiera* and *Hamiltonella* of F1 females in treatments U♀ × U♂ and U♀ × RF♂. RF: rifampicin treated whiteflies, U: untreated whiteflies. The relative density of each symbiont in the control treatment U♀ × U♂ was set to 1. Different letters above the two bars for each symbiont indicate significant difference between the two treatments based on Mann-Whitney test at α = 0.05.

For technical reasons, a separate cohort of F1 adults, which developed from rifampicin-treated adults (F0), were used in the mating experiment for F1. Both the primary and secondary symbionts were drastically reduced in both females and males produced by the rifampicin-treated F0 adults compared to those of control (Fig. 5). Compared to the control U♀ × U♂ of F1 adults, the performance of F1 and F2 whiteflies of treatment U♀ × RF♂ was largely unaffected in all performance variables examined (Table 2); similarly, both the primary and secondary symbionts in F2 females produced in treatment U♀ × RF♂ of F1 adults remained at similar levels to those of the control (Fig. 6). In contrast, the F1 females in treatments RF♀ × U♂ and RF♀ × RF♂ produced only a few eggs and all the eggs failed to hatch (Table 2).

## Discussion

Experimental techniques for manipulating the symbiont community provide opportunities to evaluate the roles of individual symbionts in a complex system. However, most of the techniques employed for this purpose, including curing by antibiotics and transfection by microinjection, are largely derived by trial-and error rather than any guiding theory or general rules. We used the antibiotic rifampicin for symbiont curing, because in previous studies of whiteflies this antibiotic was reported to provide better selective elimination of secondary symbionts than several other antibiotics^44^,^45^,^46^,^47^. During the last four years, we repeated the experiment as presented in Table 1 three times, with similar results. Consistently, we never obtained any F2 nymphs following a rifampicin treatment of F0 adults and consequently we have been unable to establish MED whitefly lines lacking *Hamiltonella* for further experimentation. Similarly, we have been unable to establish MEAM1 whitefly lines lacking a given symbiont for experimentation^56^.

The failure to establish MED whitefly lines lacking *Hamiltonella*, as reported here, is in contrast with the results reported in Su *et al.* (2013)^47^ and Xue *et al.* (2012)^46^. Like the MED whitefly tested in this study, the experimental culture of the MED whitefly examined by Su *et al.* (2013)^47^ harbours the primary symbiont *Portiera* and only one secondary symbiont *Hamiltonella*. Su *et al.* (2013)^47^ reported a MED whitefly line lacking *Hamiltonella* (named H^−^ line) was established by feeding newly emerged whitefly adults an artificial diet with 50 μg/ml rifampicin for 48 h through parafilm membrane sachets that cured *Hamiltonella* completely without affecting *Portiera*. They were then able to conduct introgression of this H^−^ line with the original MED whitefly line, which bore *Hamiltonella* (named H^+^), and establish one H^−^ and one H^+^ MED whitefly line with similar genetic backgrounds for a series of experiments to investigate the roles of *Hamiltonella*, such as virus transmission, via comparison of performance between the two MED whitefly lines. We were very interested in their success of establishing the MED whitefly H^−^ and H^+^ lines because we had repeatedly failed to do so using similar experimental procedure. We communicated with the first and corresponding author of Su *et al.* (2013)^47^, Dr. Qi Su (email: suqicaas@163.com) and Dr. Youjun Zhang (email: zhangyoujun@caas.cn). They informed us that they later detected recovery of *Hamiltonella* in their H^−^ line, and they had to treat the H^−^ line with 50 μg/ml rifampicin repeatedly to reduce the symbiont in this line for further experiments. On 30 August 2013, Dr. Youjun Zhang and Dr. Qi Su offered us whitefly samples from their H^−^ and H^+^ lines to do a molecular examination of the symbionts in these two lines. We confirmed that all individuals of both their H^−^ and H^+^ MED whitefly lines harboured *Hamiltonella*, in addition to the primary symbiont *Portiera*.

In their subsequent publications, Su *et al.* reported that *Hamiltonella* in their H^−^ MED whitefly line was not completely cured and that they had to treat the H^−^ line with rifampicin consecutively for three generations to cure the secondary symbiont^57^ and then maintain the H^−^ line for further experiments to investigate the roles of *Hamiltonella*^57^,^58^,^59^. In each of their experiments the H^−^ MED whitefly line did not receive treatment of rifampicin for three generations prior to its use in the comparative trials with the H^+^ line^57^,^58^,^59^. Despite this precaution, it is still questionable whether the differences in performance between their H^−^ and H^+^ MED whitefly lines were due to the absence or presence of *Hamiltonella* because the H^−^ line may have achieved some recovery of *Hamiltonella* in the three generations prior to the experiments. In addition antibiotic treatments may exert a range of non-specific effects on the hosts for many generations^55^.

The effects of rifampicin on the symbionts of MED reported in Ahmed *et al.* (2010)^45^ and Xue *et al.* (2012)^46^ also appear unusual. First, Ahmed *et al.* (2010)^45^ reported that all the six secondary symbionts, that had been recorded from all the species of the *B. tabaci* complex up to that time, in addition to the primary symbiont *Portiera*, were found in this MED population collected from Nantong, Jiangsu, China, yet many individuals in the population were found to harbour none of the six secondary symbionts. Second, using essentially the same experimental procedure as that of Su *et al.* (2013, 2014, 2015)^57^,^58^,^59^ but with 20 times higher a concentration of the antibiotic, i.e. 1000 μg/ml versus 50 μg/ml, Xue *et al.* (2012)^46^ achieved complete inactivation of *Wolbachia* but exerted no significant effects on the primary symbiont as well as the other five secondary symbionts including *Hamiltonella* and *Arsenophonus*. Yet, in an earlier report from the same laboratory, Ahmed *et al.* (2010)^45^ presented that treatment of a MED population from the same location with 50 μg/ml rifampicin achieved about 65% inactivation of *Wolbachia* as well as about 62% inactivation of *Arsenophonus*, the only other secondary symbiont tested at that time. These contrasting reports call for closer scrutiny.

In many studies, limited effort has been made to evaluate the generality of the selective elimination techniques using antibiotics under various conditions, such as different insect hosts and different dosages of an antibiotic, and even less effort has been devoted to monitor the changes of symbiont profiles through time following an antibiotic treatment. We used five concentrations of rifampicin and found little concentration-dependent effects of the antibiotic within the range from 1 μg/ml to 100 μg/ml. The outcome of concentration independence was somewhat surprising. Koga *et al.* (2007)^53^ found that the primary symbiont *Buchnera* in the pea aphid was always selectively eliminated by rifampicin treatment irrespective of the antibiotic dose from 10 ng per mg of aphid weight to 1000 ng per mg of aphid weight. We further monitored the changes of symbionts using qPCR through time and generations following a rifampicin treatment of the newly emerged F0 adults. The decrease of the secondary symbiont *Hamiltonella* occurred at a much faster rate than that of the primary symbiont *Portiera*: more than 8 days after treatment, the density of the primary symbiont in the adults was not significantly reduced compared to that of the control, while the density of the secondary symbiont was reduced to barely detectable levels immediately after the 48 h antibiotic treatment (Fig. 2). Such differential patterns of changes in density between the primary and secondary symbionts may produce the apparent but spurious observation that the secondary symbiont was cured without affecting the primary symbiont (e.g., Ruan *et al.*, 2006; Su *et al.*, 2013)^44^,^47^. However, the primary symbiont was significantly reduced later in the adulthood of F0 as well as in the 4^th^ instar and adult of F1, and the secondary symbiont was substantially reduced and barely detectable right from the egg to adulthood of F1 (Fig. 2). Although the F1 adults, of which both the primary and secondary symbionts were nearly completely depleted, were able to produce a few eggs, none of the F2 eggs hatched and as a result the whitefly cohorts went extinct. Chiel *et al.* (2009)^51^ reported that their attempt to eliminate the secondary symbiont *Rickettsia* from a MEAM1 culture, by either feeding the adults with various antibiotics or injecting antibiotics into adults and old nymphs, achieved no success, and as a result they were unable to establish *Rickettsia*-free whitefly lines for further experimentation. A similar phenomenon was observed in the tsetse flies, for which treatment with the antibiotic tetracycline or rifampicin eliminated all symbionts but reduced the fecundity of the treated females and made them sterile^54^. Zhong and Li (2013)^48^ reported that treatment of a MEAM1 culture with an artificial diet containing 50 μg/ml tetracycline for 48 h reduced the density of *Wolbachia* but did not achieve elimination of the symbiont.

In the pea aphid, attempts to remove the primary and certain secondary symbionts and stably establish host lines of a given symbiont have had some consistent successes^9^,^12^,^14^,^15^,^19^,^20^,^53^,^60^,^61^,^62^. The mechanism underlying the selective elimination of the various symbionts from the pea aphid is not entirely clear. Koga *et al.* (2007)^53^ speculated that the selective elimination of *Buchnera* may be associated with genome evolution of this primary symbiont, through which *Buchnera* has lost many genes for outer membrane proteins and those for the biosynthesis of lipopolysaccharides. The loss of these genes results in structurally fragile membrane in symbiont and may increase the permeability of rifampicin^63^, whereas *Serratia* has a thick outer membrane^64^. In theory, rifampicin could completely remove the primary symbiont *Portiera* from whiteflies because this antibiotic inhibits DNA-dependent RNA polymerase in bacterial cells and prevents transcription of messenger RNA. However, Ruan *et al.* (2006)^44^ found that *Portiera* was not cured, and they speculated that only a reduction of *Portiera* may have occurred after rifampicin treatment suggested a quantitative analysis of the symbiont was needed. Using both diagnostic PCR and quantitative PCR approaches, the current study showed that the density of *Portiera* was reduced after rifampicin treatment. However, the reduction of *Portiera* was more substantial in the offspring of the adults that received the antibiotic treatment than that of the antibiotic-treated adults. The reduction of *Portiera* in the adults following the treatment was not significant compared to the control until more than eight days after the treatment (Fig. 2).

Insect symbionts have been recognized as potential targets for insect pest control and evidence of negative effects of antibiotic treatment on the performance of the host insects has frequently been reported^53^,^65^,^66^,^67^. In the current study, the treatment of rifampicin exerted no significant effects on survival of the adult whiteflies; however, their offspring suffered a reduction of survival and could not produce viable offspring (Fig. 1). The mating experiments further indicated that rifampicin treatment exerted little negative effects on the adults, but had substantial negative effects on their offspring (Table 2). The negative effects of rifampicin on whitefly observed in the current study are consistent with those recorded by Ruan *et al.* (2006)^44^ and agree with the observation that symbionts are transmitted maternally^31^. The adverse effects of the rifampicin treatment would be carried over to F1 and F2 only when F0 females received rifampicin treatment regardless of the antibiotic treatment of the F0 males, i.e. rifampicin exerted its effects largely via females (Table 2). However, the potential nonspecific deleterious effects of an antibiotic is a confounding factor and is difficult to separate from the host phenotypes lacking a specific symbiont^55^. Thus, great caution must be taken when interpreting the differences of performance between host lines artificially cured of certain symbionts and the control line, especially when the comparative experiments are conducted immediately after the antibiotic treatment (e.g., Raina *et al.*, 2015)^68^.

The responses of symbionts as well as their associations with host insects to environmental stress are complex^2^,^4^,^5^,^8^,^52^. For example, Brumin *et al.* (2011)^39^ found that in an Israel population of the MEAM1 whitefly, *Rickettsia* sp. outside bacteriosomes was reduced under heat shock whereas *Portiera* and *Hamiltonella* residing inside bacteriosomes were little affected; the authors speculated that the bacteriosomes protect the symbionts. In contrast, Shan *et al.* (2014)^69^ found that in a Chinese population of MEAM1 whitefly, both *Portiera* and *Hamiltonella* in bacteriosomes were reduced after high temperature treatment whereas the scattered symbiont *Rickettsia* sp. was hardly affected. Moreover, Shan *et al.* (2014)^69^ found that following heat treatment of the whiteflies the reduction of the secondary *Hamiltonella* occurred much more quickly than that of the primary symbiont *Portiera*, a pattern similar to the differential reduction observed in the current study (Fig. 2). The complex association of symbiont community and host may be attributable to their long co-evolution history. In the whitefly *B. tabaci*, the genome of the primary symbiont *Portiera* is small apparently due to reduction via evolution, and the secondary symbiont *Hamiltonella* appears to possess genes that may be involved in nutritional metabolism^26^,^27^,^70^,^71^. However, a more recent genomic analysis on the metabolic co-evolution in the bacterial symbiosis of whiteflies indicates that the complementary relationship between *Portiera* and *Hamiltonella* may not be as direct and extensive as previously thought^28^. It was possible that the host has the priority to protect *Portiera* under disadvantageous situations because its physiological activities depend much more on the primary symbiont than on the secondary symbiont *Hamiltonella*, so that the density of *Portiera* declined at a slower pace than that of *Hamiltonella* (Fig. 2).

## Methods

### Whiteflies and plants

A colony of the MED whitefly was originally collected in 2009, in Zhejiang Province, China and GenBank accession numbers of its mitochondrial cytochrome oxidase I (*mtCOI*) is GQ371165^72^. This MED colony bears the primary symbiont *Portiera* and only one secondary symbiont *Hamiltonella*^36^. The whitefly population was cultured on cotton, *Gossypium hirsutum* (cv. Zhe-Mian 1793), plants at 26 ± 1 °C, 14L: 10D (Light 6:00–20:00), 60–80% relative humidity. The purity of the culture was monitored using the random amplified polymorphic DNA PCR (RAPD-PCR) technique and the sequence of *mtCOI* gene every 3–5 generations^73^,^74^. For each experiment, cotton plants at 5–6 true leaf stage were prepared in a greenhouse at 20–30 °C, 14L: 10D (natural lighting supplemented with artificial light), 50–70% relative humidity. Prior to a test, all leaves from each plant were checked with a 20 × hand lens to ensure that plants were insect-free.

### Antibiotic treatments

The antibiotic treatment was performed via an artificial diet-feeding approach. A glass tube (36 mm in diameter, 50 mm in length, open at both ends) was used as the feeding chamber^44^. Firstly, the tube was covered with a stretched parafilm membrane at one end, and then 0.5 ml diet solution was placed on the membrane and covered with another layer of stretched parafilm to enclose the diet solution. The antibiotic-treatedt diet solution was 5 mM phosphate buffer (pH 7.0) and 25% sucrose (W/V) with rifampicin (Sigma, no. R3501). Diet solution without rifampicin was prepared as the control. After the introduction of adult whiteflies into the feeding chamber, the chamber was sealed with gauze at the open end and the adult whiteflies could feed ad lib for 48 h. The offspring of adult whiteflies (F0) that fed on artificial diet are called F1 whiteflies, and the offspring of F1 are called F2 whiteflies. The antibiotic treatments and all experiments were performed in a climate room at 26 ± 1 °C, 14 L: 10D, 60–80% relative humidity.

### Treatment of F0 whitefly adults by rifampicin at different concentrations

F0 whitefly adults were fed with diet solutions of rifampicin at five different concentrations in addition to a control, and the curing frequencies of their primary symbiont *Portiera* and secondary symbiont *Hamiltonella* were examined after treatment (Table 1). For each of the rifampicin concentrations, approximately 100 F0 adults were introduced into a feeding chamber to feed for 48 h. Immediately after the 48 h feeding, 22 F0 female adults were randomly sampled to examine the curing frequencies of the two symbionts using a diagnostic PCR. The remaining F0 adults in each of the six feeding chambers were transferred to two clean cotton plants in a cage (50 × 50 × 50 cm) where they were maintained for mating and oviposition. On the 13^th^ day following transfer to the cotton plants, another batch of 22 F0 female adults was randomly sampled to examine the curing frequencies of the two symbionts at this time, and the rest of the F0 adults were removed from the cotton plants and discarded. After a further 17 days, when the F1 whiteflies had developed to adults, 22 females were randomly sampled to examine the curing frequencies of the two symbionts in F1. In addition, approximately 200 F1 adults including both males and females (ca. 1:1.2 ratio) were transferred to two new plants in another cage for feeding, mating and oviposition. Fourteen days later, all the F1 adults were removed from the cotton plants and their progenies (F2) were reared for further examination of symbiont curing frequencies.

### Effects of rifampicin treatment on whitefly survival and temporal dynamic of symbiont density

Since no F2 nymphs were produced from any of the rifampicin treated whitefly cohorts and the curing frequencies of symbionts between F0 and F1 whiteflies differed markedly, the survival percentages of F0 and F1whiteflies and temporal dynamics of their symbiont quantity were further examined. Since the above experiments showed little concentration effect within the range of 1–100 μg/ml, we chose the concentration of 30 μg/ml rifampicin for all the following experiments. For F0 adults, their symbiont densities were examined by quantitative PCR at different time points after rifampicin treatment, and for F1 whiteflies their symbiont densities were examined at four different development stages (Fig. 2). The protocol for rifampicin treatment of whiteflies was the same as above, i.e., approximately 100 F0 adults were introduced into a feeding chamber to feed for 48 h. Four replicates were conducted. At the end of the 48 h feeding, the dead and live adults in each of the four feeding chambers were counted, and the live adults were transferred to two clean cotton plants in a cage. Following the transfer, 12 F0 female adults were collected randomly to examine symbiont density on the 0, 3^rd^, 8^th^ and 13^th^ day respectively. To examine the change of symbiont density in F1 whiteflies, 120 rifampicin-treated F0 whitefly female and male adults (ca. 1:1.2 ratio) were introduced into six leaf-clip cages^75^, 20 adults in each cage, on two cotton plants (three clip cages on each plant) to feed and oviposit for 48 h. The F0 adults were then removed from the clip cages and discarded, and the F1 eggs were reared to adult emergence. During the development of F1 whiteflies, 12 individuals were randomly collected from the clip cages at egg, 2^nd^ instar, 4^th^ instar (pupae), and female adult stage respectively for examination of symbiont quantity. To examine the survival percentages of F1 whiteflies, 10 female and 10 male F0 adults were introduced into the same kind of clip cage to feed and oviposit for 48 h. The number of eggs on a leaf enclosed in a clip cages were counted and reared to adult emergence, and the adults were then counted to calculate the survival percentage of F1 from egg to adult. The same method was followed to examine the survival percentage of F2 whiteflies. Eight replicates (i.e. 8 clip cages) were conducted for F1 and F2 respectively.

### Mating experiments between rifampicin-treated and untreated whiteflies

To identify whether the effects of rifampicin treatment on whiteflies were associated with host sex, mating experiments of rifampicin-treated and untreated adults were conducted in F0 and F1whiteflies respectively. Virgin adults were prepared by placing single whitefly pupa into separate glass tubes (5 × 0.5 cm) prior to emergence^76^. When adults emerged, approximately 300 newly emerged females and 300 newly emerged males were respectively transferred to antibiotic treatment feeding chambers (100 adults per chamber) before mating, where the adults fed ad lib on 30 μg/ml rifampicin diet solution for 48 h or on rifampicin free diet solution for 48 h as control. Four mating treatments were conducted in F0 whiteflies: (A) U♀ × U♂, i.e. untreated female × untreated male, (B) U♀ × RF♂, i.e. untreated female × rifampicin-treated male, (C) RF♀ × U♂, i.e. rifampicin-treated female × untreated male, and (D) RF♀ × RF♂, i.e. rifampicin-treated female × rifampicin-treated male (Table 2). Eight replicates were conducted for each of the four treatments. In each replicate of a given treatment, 10 virgin females and 10 virgin males were placed in a clip cage on a cotton plant to feed, mate and oviposit for 48 h. Adults were then removed from the clip cages and discarded, and 24 untreated adults (12 females and 12 males) from treatment A and 24 rifampicin treated adults (12 females and 12 males) from treatment D were randomly collected to examin symbiont density using quantitative real-time PCR. After a further 15 days, the number of eggs and nymphs were counted in each clip-cage to examine egg hatching rate. Two days later, when the whiteflies were close to emergence, they were observed daily to record and collect newly emerged adults for calculation of the developmental duration from egg to adult and sex ratio. All the newly emerged adults were placed in −80°C upon collection each day. Emergence of F1 adults was assumed to be completed when no further emergence was observed for five consecutive days. Then 12 female adults were taken randomly from all the adults from each of the treatments for examination of their symbiont quantity using the same protocols as above.

The same mating treatments were conducted for F1 whiteflies. Firstly, to obtain F1 virgin whitefly adults, approximately 100 newly emerged adults were introduced into a feeding chamber to feed for 48 h on rifampicin diet solution or rifampicin free diet solution as control, and then the live adults were transferred to clean cotton plants for oviposition. Fifteen days later all the F0 adults were removed and discarded. When the F1 whiteflies developed to the pupal stage, newly emerged, virgin female and male adults of F1were obtained using the same method as described above. Likewise, four mating treatments of F1 were conducted: (A1) untreated female × untreated male, (B1) untreated female × rifampicin-treated male, (C1) rifampicin-treated female × untreated male, and (D1) rifampicin-treated female × rifampicin-treated male. Observations on the performance of F2 whiteflies and examination of their symbiont quantity were conducted using the same protocol as that for F1.

### DNA extraction, PCR and qPCR

Total nucleic acid was extracted following a previous description^74^. For adults and 4^th^ instar nymphs, whitefly individuals were ground in 40 μl of ice-cold lysis buffer on parafilm and then transferred into a 0.2 ml microtube. For eggs and 2^nd^ instar nymphs, 20 μl lysis buffer was used. Lysis buffer contained 5 mM of Tris-HCl (pH 8.0), 0.5 mM of EDTA, 0.5% Nonidet P-40 and 1 mg/ml of proteinase K. Extractions were incubated at 65 °C for 2 h, and then 100 °C for 10 min. After a brief centrifugation, the extractions were kept at −20 °C.

Diagnostic PCR analyses were conducted with Taq polymerase (Takara, Dalian, China) in a PTC-200 thermocycler (Bio-Rad, CA, USA)^36^. The symbionts were quantified by qPCR with the SYBR^®^ Premix Ex TaqTM (Takara, Dalian, China) and Bio-Rad CFX96TM Real-Time System (Bio-Rad, CA, USA) as described by Bing *et al.* (2013)^32^. *Portiera* and *Hamiltonella* were quantified in terms of 16 S rRNA gene. In addition, *B. tabaci β*-*actin* gene was quantified as an internal standard for normalization. All the primers are shown in Table 3.

### Statistical analysis

The data of survival percentage and sex ratio were transformed by arcsine square root to achieve normality of variances and used for significance tests, and then back-transformed for presentation. The survival percentages between untreated and rifampicin-treated whiteflies within each generation were analyzed by Student’s *t*-test (Fig. 1). The symbiont curing frequencies were analyzed by nonparametric Chi-square tests at α = 0.05 (Table 1). For the measurements of each of the biological variables observed in the mating experiments, one-way ANOVA and least-significant difference (LSD) test were applied to analyze the variation between treatments, and the Student’s *t* test was applied when data were obtained for only two treatments (Table 2). For the data on changes of density of symbionts (Figs 2, 3, 4, 5, 6), nonparametric tests were used: Kruskal-Wallis for multiple comparisons and Mann-Whitney for two sample comparisons at α = 0.05, because some of the data do not conform to a normal distribution as indicated by Shapiro-Wilk test. All the data analyses were performed using SPSS 20.0 Statistics.

## Additional Information

**How to cite this article**: Zhang, C.-R. *et al.* Differential temporal changes of primary and secondary bacterial symbionts and whitefly host fitness following antibiotic treatments. *Sci. Rep.* **5**, 15898; doi: 10.1038/srep15898 (2015).

## Figures and Tables

**Table 1:** Frequency of curing of primary symbiont *Portiera* and secondary symbiont *Hamiltonella* in F0* and F1* MED whitefly adults after the F0* adults were fed with a rifampicin diet-solution at various concentrations.

**Rifampicin concentration (μg/ml)**	N	No. of individuals with a given symbiont cured on day 0		No. of individuals with a given symbiont cured on day 13		No. of individuals with a given symbiont cured on day 30	
		*Portiera*	*Hamiltonella*	*Portiera*	*Hamiltonella*	*Portiera*	*Hamiltonella*
100	22	1 a	3 a	6 a	18 a	22 a	22 a
30	22	3 a	2 a	8 a	18 a	22 a	22 a
15	22	1 a	1 a	7 a	20 a	22 a	22 a
5	22	2 a	3 a	7 a	18 a	22 a	22 a
1	22	1 a	1 a	3 ab	19 a	22 a	20 a
0 (Control)	22	0 a	0 a	0 b	0 b	0 b	0 b

^*^F0 and F1 whitefly adults are those that we fed with a rifampicin diet solution and their offspring respectively; N: the number of whitefly adults tested by diagnostic PCR analysis; the same letters in a columns indicate non-significant difference at α = 0.05 (Chi-square tests).

**Table 2:** Effects of rifampicin treatment on whitefly fitness after mating between untreated and rifampicin-treated adults in F0 and F1 generation.

Mating treatments	Number of eggs produced per 10 females in 48 h	% egg hatching	% developing from 1^st^ instar to adult emergence	Development duration from egg to adult (days)		% adult female
				Female	Male	
F0 adults						
* *U♀ × U♂	150.0 ± 13.73 a	92.8 ± 0.77 a	95.5 ± 0.63 a	21.2 ± 0.06 c	22.1 ± 0.14 c	63.4 ± 2.95 a
* *U♀ × RF♂	163.8 ± 15.01 a	91.8 ± 1.01 a	96.3 ± 0.73 a	21.0 ± 0.09 c	21.7 ± 0.16 c	67.0 ± 2.38 a
* *RF♀ × U♂	145.3 ± 16.23 a	87.4 ± 2.17 b	95.8 ± 0.56 a	29.1 ± 0.23 a	28.9 ± 0.27 a	56.3 ± 5.66 a
* *RF♀ × RF♂	121.4 ± 14.17 a	86.3 ± 1.33 b	94.7 ± 0.41 a	26.9 ± 0.13 b	26.6 ± 0.18 b	58.6 ± 1.69 a
F1 adults						
* *U♀ × U♂	135.8 ± 11.29 a	96.9 ± 0.48 a	95.4 ± 0.71 a	22.7 ± 0.09 a	23.2 ± 0.11 a	58.6 ± 3.40 a
* *U♀ × RF♂	148.9 ± 5.56 a	96.3 ± 0.63 a	93.0 ± 0.72 b	22.6 ± 0.10 a	22.9 ± 0.10 a	62.2 ± 3.78 a
* *RF♀ × U♂	5.7 ± 1.32 b	0	—	—	—	—
* *RF♀ × RF♂	16.1 ± 1.44 b	0	—	—	—	—

*F0 and F1 whitefly adults are those that we fed with a rifampicin diet solution and their offspring respectively; RF: rifampicin-treated whitefly, U: untreated whitefly; Data are means of 8 replicates with standard errors ( ± SE); the different letters in the same column within each whitefly generation indicate significant differences based on LSD test after one-way ANOVA (*P* < 0.05); “—”: no data because none of the F2 eggs hatched.

**Table 3:** Primers used for PCR and qPCR.

Organism	Target gene	Primer name	Primer sequences (5′ → 3′)	Length (bp)	Reference
PCR					
* Portiera*	16S rRNA	Por-F Por-R	GGAAACGTACGCTAATAC TGACGACAGCCATGCAGCAC	~900	77
* Hamiltonella*	16S rRNA	Ham-F Ham-R	TGAGTAAAGTCTGGGAATCTGG AGTTCAAGACCGCAACCTC	~750	78
qPCR					
* Portiera*	16S rRNA	Port73-F Port266-R	GTGGGGAATAACGTACGG CTCAGTCCCAGTGTGGCTG	~200	79
* Hamiltonella*	16S rRNA	H-16S-Fis H-16S-Ris	GCATCGAGTGAGCACAGTTT TATCCTCTCAGACCCGCTAGA	~240	39
* B. tabaci*	*β-actin*	Actin-F Actin-R	TCTTCCAGCCATCCTTCTTG CGGTGATTTCCTTCTGCATT	~200	80
